# Vasculitis Pathogenesis: Can We Talk About Precision Medicine?

**DOI:** 10.3389/fimmu.2018.01892

**Published:** 2018-08-14

**Authors:** Seza Ozen, Ezgi Deniz Batu

**Affiliations:** ^1^Division of Rheumatology, Department of Pediatrics, Hacettepe University Faculty of Medicine, Ankara, Turkey; ^2^Division of Rheumatology, Department of Pediatrics, University of Health Sciences, Ankara Training and Research Hospital, Ankara, Turkey

**Keywords:** systemic vasculitis, genome-wide association studies, monogenic vasculitis, biomarker, precision medicine

## Abstract

Precision medicine is designing the medical care by taking into account the individual variability for each person. We have tried to address whether the existing data may guide precision medicine in primary systemic vasculitides (PSV). We have reviewed genome-wide association studies (GWAS) data, lessons from monogenic mimics of these diseases, and biomarker studies in immunoglobulin A vasculitis/Henoch–Schönlein purpura, Kawasaki disease, anti-neutrophil cytoplasmic antibody-associated vasculitis, polyarteritis nodosa (PAN), Takayasu arteritis, and Behçet’s disease (BD). GWAS provide insights about the pathogenesis of PSV while whole exome sequencing studies lead to discovery of monogenic vasculitides, phenotype of which could mimic other types of vasculitis such as PAN and BD. Monogenic vasculitides form a subgroup of vasculitis which are caused by single gene alterations and discovery of these diseases has enabled more specific therapies in these patients. With increasing number of studies on biomarkers, new targets for treatment appear and better and structured follow-up of PSV patients will become possible. Proteomics and metabolomics studies are required to better categorize our patients with PSV so that we can manage them appropriately and offer more targeted therapy.

## Introduction

Precision medicine is defined as designing the medical care for each person with optimum efficiency in prevention and treatment by considering the individual variability in genes, environment, and life style (1). Precision medicine enables to translate benchside knowledge to bedside and provide “targeted” treatment for the patient. Genetic or molecular profiling of patients is very important for precision medicine; however, there are limited data in primary systemic vasculitides (PSV).

Primary systemic vasculitides are heterogeneous. Since these are mostly rare diseases, we lack the vast translational medicine data we have in common diseases such as rheumatoid arthritis. Furthermore, controlled studies in PSV might not reflect the real-life scenarios. Thus, it is important to tailor treatment according to each patient instead of applying general recommendations which are based on controlled studies.

Thanks to improved and *cheaper* genomic techniques, we have gathered important data that may be used on the bedside. Genetic studies including mainly genome-wide association studies (GWAS) and whole exome sequencing studies have led to important discoveries in disease pathogenesis of PSV. However, we lack pharmacogenomics studies. We are also in need of more biomarker studies to provide novel candidate targets for therapy and help us to predict prognosis. Predicting poor prognosis or high relapse rate through biomarkers could guide physicians to tailor treatment according to the patient. More intensified immunosuppressive therapy could be required in patients with poor prognosis while longer maintenance therapy could prevent relapses in patients with high relapse risk.

In this article, we review the GWAS results, monogenic vasculitis, and existing biomarkers, which have implications on precision medicine in treatment of PSV. We have mainly focused on immunoglobulin A vasculitis/Henoch–Schönlein purpura (IgAV/HSP), Kawasaki disease (KD), polyarteritis nodosa (PAN), anti-neutrophil cytoplasmic antibody (ANCA)-associated vasculitis (AAV), Takayasu arteritis (TA), and Behçet’s disease (BD).

## What We Have Learned from GWAS in PSV

Genome-wide association studies provide an important step forward in our understanding of vasculitis pathogenesis. Focusing on subgroups of patients with PSV will provide further insight while profiling patients to tailor treatment individually. There are several GWAS in patients with different types of PSV such as IgAV/HSP, KD, AAV, TA, and BD (Table 1). There is no GWAS in PAN patients at present.

**Table 1:** The GWAS in primary systemic vasculitides.

Disease	Number of GWAS performed	Reference	The country of origin	Number of patients^a^	Genes found	Implications in treatment
IgAV/HSP	1	(2)	Spain	285	*HLA-DRB1* (potential relevance; not genome-wide significance)	–
KD	14	(3–16)	Taiwan, Korea, and Japan	262–1,182	*BCL2L11, KCNN2, TIAM1, NEBL, TUBA3C, PELI1, PLCB4*/*PLCB1 CRP, HLA, CD40, BLK, FCGR2A, NMNAT2, HCP5, COPB2, ERAP1, NAALADL2, ZFHX3, NFKBIL1, LTA, DAB1, IGHV*	SLC8A1 (Ca signaling pathway): use of calcineurin inhibitors in KD
AAV	3	(21–23)	European countries and UK	492–1,986	*HLA-DQ, HLA-DP, SERPINA1, PTPN22, PRTN3, SEMA6A*	–
TA	3	(24–26)	Turkey, North America, and Japan	379–693	*HLA-B/MICA, HLA-DQB1/HLA-DRB1, FCGR2A/FCGR3A, IL12B, IL6, RPS9/LILRB3*	IL6: use of anti-IL-6 drugs
						IL12B: use of therapies targeting IL-12/IL-23
BD	9	(27–35)	Turkey, UK, China, Korea, Japan, Thailand Iran, Afghanistan, Lebanon, Cape Verde, Curacao, Dominican Republic, Greece, Israel, Jordan, Morocco, and Surinam	152–3,645	*HLA class I* (especially *HLA-B51*), *IL10, IL23R-IL12RB2, ERAP1, STAT4, GIMAP, CCR1, KLRC4, FUT2, IL12A, NAALADL2, YIPF7, KIAA1529, CPVL, UBAC2, LOC100129342, UBASH3B*	IL12A, IL23R-IL12RB2: use of therapies targeting IL-12/IL-23

*AAV, ANCA-associated vasculitis; BD, Behçet’s disease; GWAS, genome-wide association studies; IgAV/HSP, immunoglobulin A vasculitis/Henoch–Schönlein purpura; KD, Kawasaki disease; TA, Takayasu arteritis; IL, interleukin; CRP, C-reactive protein*.

*^a^The minimum and maximum number of patients were shown when there are more than one GWAS*.

### Immunoglobulin A Vasculitis/Henoch–Schönlein Purpura

In the GWAS of 285 Spanish patients with IgAV/HSP, Lopez-Mejias et al. observed *p* values of potential relevance (below genome-wide significance) for the positions 11 and 13 of *HLA-DRB1* (2). This study implicated HLA Class II in the pathogenesis of the disease. Larger multinational studies may be needed to confirm this association and look for associations with certain cytokines and aberrant glycosylation of IgA1. Investigation of the association of the IgAV/HSP kidney disease with IgA nephropathy in a combined study will also be of major interest, since the IgA nephropathy has been claimed to be in the spectrum of the disease, lacking systemic features. So far, the GWAS in IgAV/HSP has not had a substantial offer to precision medicine.

### Kawasaki Disease

There are more than 10 GWAS performed in KD (3–16). The results of these GWAS may affect our medical practice since these help us to define KD patients who are at risk of developing intravenous immunoglobulin (IVIG) resistance and/or coronary involvement. The susceptibility genes associated with the development and severity of coronary involvement were mainly identified as *KCNN2, TIAM1, NEBL, TUBA3C, PELI1*, and *PLCB4*/*PLCB1* (3, 6, 7, 13, 14). Shimizu et al. performed a pathway-based association analysis on a GWAS data set to identify risk alleles for coronary artery abnormalities in KD (17). They demonstrated susceptibility variants in the SLC8A1 calcium signaling pathway which were associated with development of KD and coronary artery abnormalities (17). Their results suggested this pathway as a therapeutic target supporting the use of calcineurin inhibitors in acute KD.

Recently, Kwon et al. have performed an IVIG-response stratified GWAS to search for IVIG-response-specific genetic variants associated with KD (11). They have identified an intronic single-nucleotide polymorphism (SNP) in *BCL2L11* which was significantly associated with KD in IVIG responders but not in non-responders (11). In the first weighted genetic risk score study based on a GWAS in KD, Kuo et al. have presented the predictive model integrating the additive effects of 11 SNPs to provide a prediction for IVIG responsiveness (18). Thus these studies may be regarded to have an effect on precision medicine since they may define how we treat these patients in the coming days.

On the other hand, other GWAS have identified susceptibility genes associated with KD (mainly *HLA, CD40, BLK, FCGR2A, NMNAT2, HCP5, COPB2, ERAP1, NAALADL2, ZFHX3, NFKBIL1, LTA, DAB1, IGHV*) (4, 5, 9, 10, 12, 15, 16).

In a study of replication and meta-analysis of GWAS in KD, Chang et al. identified risk alleles confirming the importance of B lymphoid tyrosine kinase (BLK) which suggested a role for B-cell-mediated therapies in KD (19). Lv et al. reviewed the genes which had statistically significant associations with KD, from candidate gene studies and GWAS (20). They showed that cellular calcium ion hemostasis, immune and inflammatory responses were the main functional categories representing associated genes (20).

### ANCA-Associated Vasculitis

The GWAS of AAV has indeed provided quite important data on the genetic disparities of these diseases. In the first GWAS in AAV, Lyons et al. demonstrated that granulomatous polyangiitis (GPA, Wegener’s) and microscopic polyangiitis were genetically distinct diseases and anti-myeloperoxidase ANCA was associated with *HLA-DQ* while anti-proteinase 3 (anti-PR3) ANCA was associated with *HLA-DP, SERPINA1*, and *PRTN3* (21). This study has revealed the antigenic specificity of ANCA forms the strongest associations in AAV. Xie et al. identified risk variants for GPA in *SEMA6A* and *HLADP* (22). Thus, these GWAS have not only had an impact on our vasculitis classification but also provided us data on how to predict the course in these patients.

On the other hand, Merkel et al. identified genome-wide significant associations for AAV at the *HLA-DPB1, SERPINA1, PTPN22*, and *PRTN3* loci with the largest effect coming from *HLA-DPB1* polymorphisms (23).

### Takayasu Arteritis

In a GWAS of TA patients from Turkey and North America, Saruhan-Direskeneli et al. identified susceptibility loci as *HLA-B/MICA, HLA-DQB1/HLA-DRB1*, and *FCGR2A/FCGR3A* (24). They also identified additional association effects of *PSMG1, IL12*, and *IL23* that could not reach genome-wide significance. The association with *IL12B* variants were confirmed in Japanese TA patients in a genome scanning study (25). In the most recent GWAS of TA, Renauer et al. identified susceptibility variations for TA at *IL6, RPS9/LILRB3*, and an intergenic locus on chromosome 21q22 (26). Two of these loci, *IL6* and *RPS9/LILRB3* have roles in immunoregulatory pathways which could enlighten the immunopathogenesis of TA. This may be reflecting our bedside experience for the use of anti-interleukin-6 (IL-6) in TA patients and thus serve as an example for precision medicine if we could have genotyped all these patients. On the other hand, blocking IL-12/IL-23 pathway may also be an option for selected patients.

### Behçet’s Disease

There are nine GWAS performed in BD patients that have indeed served us to understand the pathogenesis of the disease (27–35). These studies have shown genome-wide significant associations with *HLA class I* (especially *HLA-B51*), *IL10, IL23R-IL12RB2, ERAP1, STAT4, GIMAP, CCR1, KLRC4, FUT2, IL12A, NAALADL2, YIPF7, KIAA1529, CPVL, LOC100129342, UBASH3B*, and *UBAC2* regions in BD (27–35). It is important to note that endoplasmic reticulum aminopeptidase 1 (ERAP1) is an endoplasmic reticulum protein functioning as an aminopeptidase trimming peptides for loading onto MHC Class I (36). It has been demonstrated that ERAP1 contributed to the risk for BD in HLA-B51 positive individuals (31). The variants identified in BD GWAS suggest defects in pathways of adaptive and innate immune responses, sensing/processing of microbial and danger signals, and inflammatory pathways shared with spondyloarthropathies (37). These studies have suggested that the improper folding of HLA-B51 is to trigger endoplasmic reticulum stress leading to the IL-12/IL-23 pathway activation. Bakir-Gungor et al. performed a pathway analysis using BD GWAS data from two populations and demonstrated that shared pathways were mitogen-activated protein kinase signaling, transforming growth factor β signaling, focal adhesion, extracellular matrix–receptor interaction, complement and coagulation cascades, and proteasome pathways (38). Thus, GWAS in BD have mainly provided us answers for the pathogenesis of the disease. However, blocking IL-12/IL-23 pathway is being considered in light of these findings. Whether the benchside will hold true *in vivo* remains to be seen.

All these GWAS performed in PSV patients points to certain mechanisms in the pathogenesis. We clearly lack data on pharmacogenomics. And we need larger cohort of patients who are profiled genetically and phenotypically, to identify the pathogenesis and possibly the disease course and highlight treatment options.

## Can Monogenic Vasculitides Teach us Anything?

Most of our common rheumatic diseases are multifactorial diseases with the contribution of certain SNPs (as described above) for disease risk. These SNPs have a rather small impact or introduce a small risk factor for the occurrence of that vasculitis. However, in recent years we have become aware of single gene defects that have a major impact in the inflammatory pathway, causing a phenotype often mimicking a well-known vasculitis (39). Description of monogenic vasculitides has provided novel insights into disease pathogenesis and pathways of inflammation in general. On the other hand, they have enabled targeted therapies in these single-gene disorders. Deficiency of adenosine deaminase 2 (DADA2), stimulator of interferon genes (STING)-associated vasculopathy with onset in infancy (SAVI), and haploinsufficiency of A20 (HA20) are the recently defined monogenic vasculitides. Indeed, the association of monogenic complement deficiencies with systemic lupus erythematosus had taught us the role of complement in lupus pathogenesis. Similarly, the affected pathways in these diseases may enable us to design more targeted therapies in vasculitides. For example, one may need to concentrate more on the nuclear factor-κB (NF-κB) pathway or the role of macrophages.

### Deficiency of Adenosine Deaminase 2

Deficiency of adenosine deaminase 2 is associated with *CECR1* mutations and causes a PAN-like vasculopathy and autoinflammatory features (40). ADA2 is thought to trigger the macrophages to have an inflammatory phenotype and endothelial integrity is compromised in DADA2 (41). As a result of these, vasculopathy and inflammation occur.

The phenotype mimics PAN with the presence of aneurysms in visceral arteries. Clinical presentation is a spectrum from only cutaneous lesions to full blown systemic disease (40–42). DADA2 patients may also present with only hematological features such as pure red cell anemia (43). Anti-tumor necrosis factor (anti-TNF) drugs are effective in the treatment of patients with vasculitis whereas response to conventional treatment is poor. Hematopoietic stem cell transplantation is needed in severe cases (40, 44, 45). Hematopoietic stem cell transplantation should be especially considered in DADA2 patients with bone marrow failure who display predominant hematological features (46).

### STING-Associated Vasculopathy With Onset in Infancy

Stimulator of interferon genes-associated vasculopathy with onset in infancy is a type I interferonopathy caused by gain-of-function mutations in *TMEM173* encoding for STING (47). STING hyperfunction results in constitutive transcription of type I interferons (IFNs) which bind to type I IFN receptors and signal through Janus activating kinase/signal transducer and activator of transcription (JAK/STAT) pathway (47, 48).

Phenotype of SAVI patients could resemble GPA with severe cutaneous vasculopathy, pulmonary involvement, and ANCA positivity in some patients (49). Recently, Sanchez et al. have shown improvement in SAVI patients with baricitinib which is a selective JAK1 and JAK2 inhibitor (50).

### Haploinsufficiency of A20

Haploinsufficiency of A20 is a recently defined autosomal dominant autoinflammatory syndrome resembling BD, caused by loss-of-function mutations in *TNFAIP3* encoding for A20 protein (51). Restriction of A20 function augments NF-κB signaling which makes NF-κB-dependent proinflammatory cytokines such as TNF-α, IFN-γ, IL-17, IL-1β potential targets for treatment in these patients. Around 50 HA20 patients have been reported so far with early-onset recurrent mucosal ulcers resembling BD being the hallmark feature in most (51–61). HA20 is classified as a monogenic vasculitis here since it resembles BD; however, it is important to note that the phenotype is very heterogeneous and it may mimic autoimmune diseases such as systemic lupus erythematosus and autoimmune lymphoproliferative syndrome. Furthermore, the evidence for vasculitis in tissue samples is deficient in most patients (52).

### Familial Mediterranean Fever (FMF)-Associated Vasculitides

Familial Mediterranean fever is the most common autoinflammatory disease characterized by fever and polyserositis attacks and caused by *MEFV* mutations (62). The mutations in this gene are associated with increased IL-1 production. Colchicine is the mainstay of FMF treatment (62). Certain vasculitides are more frequent in FMF patients than normal population (63). The most common vasculitis associated with FMF is IgAV/HSP, present in around 3% of FMF patients (63, 64). Another form of vasculitis is PAN which is also more common among FMF patients when compared to the expected frequency: almost 1% of FMF patients had PAN with distinctive features such as perinephric hematoma, severe myalgia, markedly high acute phase reactants, younger age, and better survival than classic PAN (39, 63, 65). In the eastern Mediterranean where the disease is frequent, physicians should ask for symptoms of FMF in patients with IgAV/HSP and possibly PAN. Concomitant occurrence of BD and FMF was also much higher than expected in several studies and a high frequency of *MEFV* mutations was reported in BD patients (66–68). In addition, a meta-analysis has confirmed the association between *MEFV* mutations M694V and M680I with BD (69). Other PSV such as AAV and TA may accompany FMF, as well; however, their association is not as apparent as the aforementioned diseases.

The association of PSV with this monogenic disease affects the way we treat these patients: colchicine needs to be initiated for FMF in addition to the conventional treatment of vasculitis in these patients. Anti-IL-1 therapies could be considered in resistant cases (62).

## Biomarkers in PSV

A biomarker is defined as an objectively measured characteristic marker which is evaluated as an indicator of normal biological or pathogenic processes or pharmacological responses to a therapeutic intervention (70). Biomarkers may be important for predicting the tendency to have the disease, disease activity, therapeutic options, disease flare, and disease course. For precision medicine, important biomarkers are the ones which guide us through choosing therapeutic options or determining patients with poor prognosis who need more aggressive treatment. Although there are recent studies addressing novel biomarkers, biomarker-driven treatment algorithms are not available in PSV. Furthermore, it is important to note that most of the existing biomarker studies in PSV involve mainly adult patients. Thus, the rheumatology community needs sophisticated work of proteomics and metabolomics to define the important pathways and biomarkers for our management of the patients. The main biomarkers that serve therapeutic targets in PSV are summarized in Table 2.

**Table 2:** The main biomarkers that serve targets for treatment in primary systemic vasculitides.

Disease	Biomarker	Treatment	Reference
IgAV/HSP	Factor XIII	Factor XIII concentrate to improve gastrointestinal complaints	(79, 80)
AAV	Neutrophil microparticles	Plasma exchange treatment	(103)
	Endothelial microparticles		
AAV	Complement pathway proteins	Selective C5a receptor inhibitor in replacing high-dose corticosteroids	(107, 108)
TA	IL-6	Anti-IL-6 treatment	(117, 120, 121, 123)
TA	TNF-α	Anti-TNF treatment	(121, 124)
BD	JAK/STAT	JAK inhibitors	(129)
BD	A variety of cytokines (TNF, IL-1, IL-6, IL-12/IL-23)	Cytokine targeting therapies (anti-TNF, anti-IL-1, anti-IL-6, anti-IL-17/IL-23)	(131–138)

*AAV, ANCA-associated vasculitis; BD, Behçet’s disease; IgAV/HSP, immunoglobulin A vasculitis/Henoch–Schönlein purpura; IL, interleukin; JAK, janus kinase; KD, Kawasaki disease; STAT, signal transducer and activator of transcription; TA, Takayasu arteritis; TNF, tumor necrosis factor*.

### Immunoglobulin A Vasculitis/Henoch–Schönlein Purpura

Biomarker studies in IgAV/HSP have generally focused on finding non-invasive markers for diagnosis/prediction of nephritis and renal prognosis. A few studies are present on biomarkers of prognosis for gastrointestinal involvement, as well. Sun et al. have recently identified biomarkers by a combined clinical and metabolomics analysis in children with IgAV/HSP (71). They have shown that (s)-3-hydroxyisobutyric acid, p-Cresol sulfate, and 3-carboxy-4-methyl-5-pentyl-2-furanpropanoic acid were associated with kidney involvement in IgAV/HSP. These biomarkers allowed prediction of IgAV/HSP nephritis with high sensitivity (94.7%) and specificity (80.8%) when combined with D-dimer (71). Berthelot et al. have studied the value of biomarkers for predicting the outcome of IgAV/HSP nephritis in an adult prospective cohort (72). They have demonstrated that serum Gd-IgG1, urinary IgA, IgG, IgM, neutrophil gelatinase-associated lipocalin, IL-1β, IL-6, IL-8, IL-10, IgA-IgG, and IgA-sCD89 complexes were associated with nephritis while urine IgA at disease onset could predict poor renal outcome in IgAV/HSP patients (72). Other biomarkers such as matrix metalloproteinase 9 (MMP-9), red blood cell distribution width, pentraxin 3, alpha-smooth muscle actin, and c-Met were also reported to be associated with the risk of nephritis in IgAV/HSP (73–76). As to the genetic factors, two recent studies have shown the association of inducible nitric oxide synthase (*iNOS*) gene and *IL1β* gene polymorphisms with kidney involvement in IgAV/HSP (77, 78). Thus, these biomarkers may guide us in how we manage these patients in the coming days.

Decreased factor XIII activity was suggested as a prognostic biomarker for severe gastrointestinal system involvement in IgAV/HSP patients (79, 80). Administration of factor XIII concentrate lead to improvement of gastrointestinal complaints in anecdotal case reports (80).

### Kawasaki Disease

Biomarker studies in KD are mainly focused on predicting patients who will not respond to IVIG treatment. A more intensified treatment with corticosteroids along with IVIG could be administered to the patients with high risk of IVIG unresponsiveness. Early use of biologics may also be indicated in selected cases. Elevated levels of IL-17A, IL-10 (81), ferritin (82), tenascin C (83), IL-6, C-reactive protein (CRP), percentage of circulating neutrophils (84), increased QT interval dispersion (85), and increased ratio of CD8+ HLA-DR+ T cells/CD8+ CD69+ T cells (86) are main biomarkers reported to be used for predicting IVIG resistance in KD. Validation studies are required to use these biomarkers in daily clinical practice.

### Polyarteritis Nodosa

There have been few studies on biomarkers related to disease activity in PAN. Several biomarkers such as D-dimer, anti-moesin antibody, and anti-endothelial antibodies have been associated with disease activity (87–89). However, none of these are currently being used in routine medical practice in the management of patients with PAN.

### ANCA-Associated Vasculitis

In AAV, ANCAs are the most commonly studied biomarkers. These are mainly diagnostic biomarkers and their use for monitoring disease activity is controversial. However, recent studies have suggested that especially PR3-ANCAs could be used to predict relapse in AAV (90–94). Besides ANCA, Kemna et al. have demonstrated that galactosylation and sialylation levels of IgG could predict relapse in PR3-AAV patients (95). In the recent targeted proteomics study, Ishizaki et al. have demonstrated the effectiveness of tissue inhibitor of metalloproteinase (TIMP1) as a disease activity marker for AAV and they have identified transketolase and CD92 as novel markers for evaluation of renal involvement and renal outcome in AAV (96). These may serve as valuable markers in our clinical practice. Two different proteomics studies demonstrated that serum proteomic profile differed between active systemic versus remitting patients with GPA (97, 98).

McKinney et al. studied gene-expression-based biomarkers in AAV and demonstrated that the poor diagnostic group were defined mainly by the IL7R pathway and T cell receptor signaling genes which were expressed by T cells (99). Their results also suggested that measuring the expression of only three genes; *ITGA2, NOTCH1*, and *PTPN22* could be used to define prognostic subgroups in AAV. These results raise the prospect of precision medicine in AAV, as the authors have concluded. In another study of the same group on gene-expression biomarkers, they demonstrated an association between T cell exhaustion and poor prognosis in AAV and suggested that this process could be targeted in AAV treatment (100).

As to biomarkers for response to treatment in AAV, Unizony et al. demonstrated that patients with PR3-AAV responded better to rituximab than to traditional induction/maintenance treatment with cyclophosphamide and azathioprine and suggested that an ANCA-based classification might guide immunosuppressive treatment in AAV (101). Haubitz et al. showed that immunosuppressive treatment in AAV changes the urine proteome toward remission (102).

Neutrophil microparticles (NMPs) and neutrophil extracellular traps (NETs) are biomarkers that could be targets for treatment in AAV and maybe other vasculitides as well. NMPs are membrane vesicles that induce endothelial damage in AAV, released from neutrophils upon activation by ANCA (103). Hong et al. have demonstrated more NMPs in the plasma of children with AAV than patients with inactive vasculitis and healthy controls (103). AAV patients with increased NMPs might benefit from plasma exchange therapy (103). In the same lines, endothelial MPs, important biomarkers of endothelial injury, may be important targets to be removed by plasma exchange in AAV (103).

Neutrophil extracellular traps, composed of DNA, histones, and neutrophil proteins, are released by neutrophils under the influence of inflammatory stimuli (104). Kessenbrock et al. reported NET deposition in inflamed kidneys of AAV patients (105) while Wang et al. showed that circulating NETs did not differ between patients with active vasculitis and patients in remission (106). NETs could represent a novel target for therapy in AAV; however, further studies are required to determine the exact role of NETs in AAV pathogenesis.

Recent studies have highlighted the role of complement alternative pathway activation in AAV pathogenesis (107). In a recent randomized trial, Jayne et al. have demonstrated that avacopan, an orally administered selective C5a receptor inhibitor was effective in replacing high-dose corticosteroid treatment in AAV (108).

There are other biomarkers such as macrophage migration inhibitory factor, delta neutrophil index, mean platelet volume, rheumatoid factor, and serum ferritin most of which have been recently reported to be associated with disease activity in AAV (109–113). However, further validation studies are required for these biomarkers to be commonly used while profiling AAV patients with regards to disease course and prognosis.

### Takayasu Arteritis

Biomarker studies in TA are mainly focused on differentiating active disease from inactive disease. Different biomarkers such as erythrocyte sedimentation rate (ESR), CRP, IL-2, IL-3, IL-4, IL-6, IL-8, TNF-α, IFN-γ, MMPs, TIMP1, vascular cell adhesion molecules, RANTES (Regulated on Activation, Normal T Cell Expressed and Secreted), and pentraxin 3 were associated with TA disease activity (114–122); however, none of them is validated for predicting outcome and only ESR and CRP are available in routine clinical practice. Some of the aforementioned biomarkers such as IL-6 and TNF-α serve targets for effective therapies in TA (123, 124). Goel et al. have recently demonstrated that myeloid-related protein 8/14 (MRP8/14) (S100A8/S100A9) levels were higher in patients with active disease than those with stable disease and change in MRP8/14 levels was significantly associated with the disease activity assessed by Indian TA Activity Score (125). Furthermore, MRP8/14 levels decreased significantly in responders during follow-up. They also showed that MRP8/14 was a better disease activity biomarker than ESR and nearly similar to CRP in this aspect in TA (125).

As genetic factors, a recently studied biomarker in TA, is human leukocyte antigen E (126). Goel et al. demonstrated that soluble HLA-E levels increased more frequently in TA patients with a persistently active, relapsing course than those with a persistent stable course (126). HLA-Bw52 was previously shown to be associated with higher incidence of cardiovascular events and poorer prognosis in TA patients (127). Terao et al. showed that combination of SNPs on *IL12B* and *HLA-B52:01* was significantly associated with severity of aortic regurgitation, a severe complication of TA (25).

Imaging modalities such as computed tomography, magnetic resonance imaging, and positron emission tomography are important to detect vasculitic lesions in large vessel vasculitis and in practice are used as outcome tools.

### Behçet’s Disease

Lots of disease activity biomarkers have been reported in BD. None of these (except ESR and CRP as acute phase reactants) are currently being used in routine clinical practice to profile BD patients. However, several of these biomarkers may be targeted in BD treatment. Sadeghi et al. have recently studied the serum profiles of cytokines in BD patients and demonstrated significant elevation of IL-2 in patients with uveitis (compared to recovered patients or those without uveitis) (128). The authors have thus concluded that IL-2 may be a new target for treatment of refractory BD uveitis. Tulunay et al. demonstrated that JAK1/STAT3 signaling pathway was activated in BD, possibly through activation of Th1/Th17-type cytokines such as IL-2, IL-6, IL-17, IL-23, and IFN-γ (129). They suggested that ustekinumab (anti-IL-12/IL-23) and tofacitinib (inhibiting JAK1/3) could be novel therapeutic options for BD. On the other hand, several genetic associations have also been identified in BD, including mainly the genes encoding for HLA-B51, IL-6, IL-10, IL-1β, IL-12R/IL-23R, intracellular adhesion molecule, nitric oxide, chemokine receptor type 5, toll-like receptors, and fucosyltransferase 2 (130). In the same lines, elevated levels of several proinflammatory cytokines including IL-1, IL-6, IL-17, and IL-23 have been demonstrated in patients with BD (131–137). Some of the pathways including these cytokines have been targeted successfully with biologic drugs such as etanercept (anti-TNF), infliximab (anti-TNF), tocilizumab (anti-IL-6), secukinumab (targeting IL-23/IL-17 pathway), and canakinumab (anti-IL-1) (130, 138).

## Conclusion

Precision medicine is our new aim in clinics and translational medicine will surely guide this practice. With the recent genetic studies and promising biomarker discoveries, precision medicine will be possible in PSV. The existing data needs to be confirmed in large, multicenter studies. Further proteomics and metabolomics data enlightening the involved pathways are needed. These further studies are required to profile vasculitis patients better to tailor treatment individually.

## Author Contributions

EB prepared the first draft of the article. SO made the critical revision of the article. Both authors have seen and approved the final version of the manuscript.

## Conflict of Interest Statement

SO is receiving consultancy fees from Novartis and Eczacibasi. EB declares no conflict of interest.

## References

[1] NIH. The Definition of Precision Medicine. (2018). Available from: https://ghr.nlm.nih.gov (Accessed: April 26, 2018).

[2] Lopez-Mejias R Carmona FD Castaneda S Genre F Remuzgo-Martinez S Sevilla-Perez B A genome-wide association study suggests the HLA Class II region as the major susceptibility locus for IgA vasculitis. Sci Rep (2017) 7(1):5088. 10.1038/s41598-017-03915-2 28698626 PMC5506002

[3] Kuo HC Li SC Guo MM Huang YH Yu HR Huang FC Genome-wide association study identifies novel susceptibility genes associated with coronary artery aneurysm formation in Kawasaki disease. PLoS One (2016) 11(5):e0154943. 10.1371/journal.pone.0154943 27171184 PMC4865092

[4] Burgner D Davila S Breunis WB Ng SB Li Y Bonnard C A genome-wide association study identifies novel and functionally related susceptibility loci for Kawasaki disease. PLoS Genet (2009) 5(1):e1000319. 10.1371/journal.pgen.1000319 19132087 PMC2607021

[5] Khor CC Davila S Breunis WB Lee YC Shimizu C Wright VJ Genome-wide association study identifies FCGR2A as a susceptibility locus for Kawasaki disease. Nat Genet (2011) 43(12):1241–6. 10.1038/ng.981 22081228

[6] Kim JJ Hong YM Sohn S Jang GY Ha KS Yun SW A genome-wide association analysis reveals 1p31 and 2p13.3 as susceptibility loci for Kawasaki disease. Hum Genet (2011) 129(5):487–95. 10.1007/s00439-010-0937-x 21221998

[7] Kim JJ Park YM Yoon D Lee KY Seob Song M Doo Lee H Identification of KCNN2 as a susceptibility locus for coronary artery aneurysms in Kawasaki disease using genome-wide association analysis. J Hum Genet (2013) 58(8):521–5. 10.1038/jhg.2013.43 23677057

[8] Kim JJ Yun SW Yu JJ Yoon KL Lee KY Kil HR Common variants in the CRP promoter are associated with a high C-reactive protein level in Kawasaki disease. Pediatr Cardiol (2015) 36(2):438–44. 10.1007/s00246-014-1032-1 25266886

[9] Kim JJ Yun SW Yu JJ Yoon KL Lee KY Kil HR A genome-wide association analysis identifies NMNAT2 and HCP5 as susceptibility loci for Kawasaki disease. J Hum Genet (2017) 62(12):1023–9. 10.1038/jhg.2017.87 28855716

[10] Kwon YC Kim JJ Yun SW Yu JJ Yoon KL Lee KY Male-specific association of the FCGR2A His167Arg polymorphism with Kawasaki disease. PLoS One (2017) 12(9):e0184248. 10.1371/journal.pone.0184248 28886140 PMC5590908

[11] Kwon YC Kim JJ Yun SW Yu JJ Yoon KL Lee KY BCL2L11 is associated with Kawasaki disease in intravenous immunoglobulin responder patients. Circ Genom Precis Med (2018) 11(2):e002020 10.1161/CIRCGEN.117.002020 29453247

[12] Lee YC Kuo HC Chang JS Chang LY Huang LM Chen MR Two new susceptibility loci for Kawasaki disease identified through genome-wide association analysis. Nat Genet (2012) 44(5):522–5. 10.1038/ng.2227 22446961

[13] Lin MT Hsu CL Taiwan Pediatric CVG Chen PL Yang WS Wang JK A genome-wide association analysis identifies novel susceptibility loci for coronary arterial lesions in patients with Kawasaki disease. Transl Res (2013) 161(6):513–5. 10.1016/j.trsl.2013.02.002 23454411

[14] Lin YJ Chang JS Liu X Tsang H Chien WK Chen JH Genetic variants in PLCB4/PLCB1 as susceptibility loci for coronary artery aneurysm formation in Kawasaki disease in Han Chinese in Taiwan. Sci Rep (2015) 5:14762. 10.1038/srep14762 26434682 PMC4593004

[15] Onouchi Y Ozaki K Burns JC Shimizu C Terai M Hamada H A genome-wide association study identifies three new risk loci for Kawasaki disease. Nat Genet (2012) 44(5):517–21. 10.1038/ng.2220 22446962

[16] Tsai FJ Lee YC Chang JS Huang LM Huang FY Chiu NC Identification of novel susceptibility loci for Kawasaki disease in a Han Chinese population by a genome-wide association study. PLoS One (2011) 6(2):e16853. 10.1371/journal.pone.0016853 21326860 PMC3033903

[17] Shimizu C Eleftherohorinou H Wright VJ Kim J Alphonse MP Perry JC Genetic variation in the SLC8A1 calcium signaling pathway is associated with susceptibility to Kawasaki disease and coronary artery abnormalities. Circ Cardiovasc Genet (2016) 9(6):559–68. 10.1161/CIRCGENETICS.116.001533 27879314

[18] Kuo HC Wong HS Chang WP Chen BK Wu MS Yang KD Prediction for intravenous immunoglobulin resistance by using weighted genetic risk score identified from genome-wide association study in Kawasaki disease. Circ Cardiovasc Genet (2017) 10(5):e001625. 10.1161/CIRCGENETICS.116.001625 29025760 PMC5647111

[19] Chang CJ Kuo HC Chang JS Lee JK Tsai FJ Khor CC Replication and meta-analysis of GWAS identified susceptibility loci in Kawasaki disease confirm the importance of B lymphoid tyrosine kinase (BLK) in disease susceptibility. PLoS One (2013) 8(8):e72037. 10.1371/journal.pone.0072037 24023612 PMC3758326

[20] Lv YW Wang J Sun L Zhang JM Cao L Ding YY Understanding the pathogenesis of Kawasaki disease by network and pathway analysis. Comput Math Methods Med (2013) 2013:989307. 10.1155/2013/989307 23533546 PMC3606754

[21] Lyons PA Rayner TF Trivedi S Holle JU Watts RA Jayne DR Genetically distinct subsets within ANCA-associated vasculitis. N Engl J Med (2012) 367(3):214–23. 10.1056/NEJMoa1108735 22808956 PMC3773907

[22] Xie G Roshandel D Sherva R Monach PA Lu EY Kung T Association of granulomatosis with polyangiitis (Wegener’s) with HLA-DPB1*04 and SEMA6A gene variants: evidence from genome-wide analysis. Arthritis Rheum (2013) 65(9):2457–68. 10.1002/art.38036 23740775 PMC4471994

[23] Merkel PA Xie G Monach PA Ji X Ciavatta DJ Byun J Identification of functional and expression polymorphisms associated with risk for antineutrophil cytoplasmic autoantibody-associated vasculitis. Arthritis Rheumatol (2017) 69(5):1054–66. 10.1002/art.40034 28029757 PMC5434905

[24] Saruhan-Direskeneli G Hughes T Aksu K Keser G Coit P Aydin SZ Identification of multiple genetic susceptibility loci in Takayasu arteritis. Am J Hum Genet (2013) 93(2):298–305. 10.1016/j.ajhg.2013.05.026 23830517 PMC3738826

[25] Terao C Yoshifuji H Kimura A Matsumura T Ohmura K Takahashi M Two susceptibility loci to Takayasu arteritis reveal a synergistic role of the IL12B and HLA-B regions in a Japanese population. Am J Hum Genet (2013) 93(2):289–97. 10.1016/j.ajhg.2013.05.024 23830516 PMC3738822

[26] Renauer PA Saruhan-Direskeneli G Coit P Adler A Aksu K Keser G Identification of susceptibility loci in IL6, RPS9/LILRB3, and an intergenic locus on chromosome 21q22 in Takayasu arteritis in a genome-wide association study. Arthritis Rheumatol (2015) 67(5):1361–8. 10.1002/art.39035 25604533 PMC4414813

[27] Fei Y Webb R Cobb BL Direskeneli H Saruhan-Direskeneli G Sawalha AH Identification of novel genetic susceptibility loci for Behcet’s disease using a genome-wide association study. Arthritis Res Ther (2009) 11(3):R66 10.1186/ar2695 19442274 PMC2714112

[28] Hou S Yang Z Du L Jiang Z Shu Q Chen Y Identification of a susceptibility locus in STAT4 for Behcet’s disease in Han Chinese in a genome-wide association study. Arthritis Rheum (2012) 64(12):4104–13. 10.1002/art.37708 23001997

[29] Kappen JH Medina-Gomez C van Hagen PM Stolk L Estrada K Rivadeneira F Genome-wide association study in an admixed case series reveals IL12A as a new candidate in Behcet disease. PLoS One (2015) 10(3):e0119085 10.1371/journal.pone.0119085 25799145 PMC4370488

[30] Kim SW Jung YS Ahn JB Shin ES Jang HW Lee HJ Identification of genetic susceptibility loci for intestinal Behcet’s disease. Sci Rep (2017) 7:39850 10.1038/srep39850 28045058 PMC5206652

[31] Kirino Y Bertsias G Ishigatsubo Y Mizuki N Tugal-Tutkun I Seyahi E Genome-wide association analysis identifies new susceptibility loci for Behcet’s disease and epistasis between HLA-B*51 and ERAP1. Nat Genet (2013) 45(2):202–7. 10.1038/ng.2520 23291587 PMC3810947

[32] Lee YJ Horie Y Wallace GR Choi YS Park JA Choi JY Genome-wide association study identifies GIMAP as a novel susceptibility locus for Behcet’s disease. Ann Rheum Dis (2013) 72(9):1510–6. 10.1136/annrheumdis-2011-200288 23041938

[33] Mizuki N Meguro A Ota M Ohno S Shiota T Kawagoe T Genome-wide association studies identify IL23R-IL12RB2 and IL10 as Behcet’s disease susceptibility loci. Nat Genet (2010) 42(8):703–6. 10.1038/ng.624 20622879

[34] Remmers EF Cosan F Kirino Y Ombrello MJ Abaci N Satorius C Genome-wide association study identifies variants in the MHC class I, IL10, and IL23R-IL12RB2 regions associated with Behcet’s disease. Nat Genet (2010) 42(8):698–702. 10.1038/ng.625 20622878 PMC2923807

[35] Xavier JM Shahram F Sousa I Davatchi F Matos M Abdollahi BS FUT2: filling the gap between genes and environment in Behcet’s disease? Ann Rheum Dis (2015) 74(3):618–24. 10.1136/annrheumdis-2013-204475 24326010

[36] Haroon N Inman RD. Endoplasmic reticulum aminopeptidases: biology and pathogenic potential. Nat Rev Rheumatol (2010) 6(8):461–7. 10.1038/nrrheum.2010.85 20531381

[37] Gul A Genetics of Behcet’s disease: lessons learned from genomewide association studies. Curr Opin Rheumatol (2014) 26(1):56–63. 10.1097/BOR.0000000000000003 24257369

[38] Bakir-Gungor B Remmers EF Meguro A Mizuki N Kastner DL Gul A Identification of possible pathogenic pathways in Behcet’s disease using genome-wide association study data from two different populations. Eur J Hum Genet (2015) 23(5):678–87. 10.1038/ejhg.2014.158 25227143 PMC4402634

[39] Ozen S. The changing face of polyarteritis nodosa and necrotizing vasculitis. Nat Rev Rheumatol (2017) 13(6):381–6. 10.1038/nrrheum.2017.68 28490787

[40] Navon Elkan P Pierce SB Segel R Walsh T Barash J Padeh S Mutant adenosine deaminase 2 in a polyarteritis nodosa vasculopathy. N Engl J Med (2014) 370(10):921–31. 10.1056/NEJMoa1307362 24552285

[41] Zhou Q Yang D Ombrello AK Zavialov AV Toro C Zavialov AV Early-onset stroke and vasculopathy associated with mutations in ADA2. N Engl J Med (2014) 370(10):911–20. 10.1056/NEJMoa1307361 24552284 PMC4193683

[42] Garg N Kasapcopur O Foster J II Barut K Tekin A Kizilkilic O Novel adenosine deaminase 2 mutations in a child with a fatal vasculopathy. Eur J Pediatr (2014) 173(6):827–30. 10.1007/s00431-014-2320-8 24737293

[43] Hashem H Egler R Dalal J. Refractory pure red cell aplasia manifesting as deficiency of adenosine deaminase 2. J Pediatr Hematol Oncol (2017) 39(5):e293–6. 10.1097/MPH.0000000000000805 28230570

[44] Batu ED Karadag O Taskiran EZ Kalyoncu U Aksentijevich I Alikasifoglu M A case series of adenosine deaminase 2-deficient patients emphasizing treatment and genotype-phenotype correlations. J Rheumatol (2015) 42(8):1532–4. 10.3899/jrheum.150024 26233953

[45] Van Eyck L Liston A Wouters C Mutant ADA2 in vasculopathies. N Engl J Med (2014) 371(5):478–81. 10.1056/NEJMc1405506 25075846

[46] Hashem H Kumar AR Muller I Babor F Bredius R Dalal J Hematopoietic stem cell transplantation rescues the hematological, immunological, and vascular phenotype in DADA2. Blood (2017) 130(24):2682–8. 10.1182/blood-2017-07-798660 28974505 PMC5731089

[47] Liu Y Jesus AA Marrero B Yang D Ramsey SE Sanchez GAM Activated STING in a vascular and pulmonary syndrome. N Engl J Med (2014) 371(6):507–18. 10.1056/NEJMoa1312625 25029335 PMC4174543

[48] Schneider WM Chevillotte MD Rice CM. Interferon-stimulated genes: a complex web of host defenses. Annu Rev Immunol (2014) 32:513–45. 10.1146/annurev-immunol-032713-120231 24555472 PMC4313732

[49] Munoz J Rodiere M Jeremiah N Rieux-Laucat F Oojageer A Rice GI Stimulator of interferon genes-associated vasculopathy with onset in infancy: a mimic of childhood granulomatosis with polyangiitis. JAMA Dermatol (2015) 151(8):872–7. 10.1001/jamadermatol.2015.0251 25992765

[50] Sanchez GAM Reinhardt A Ramsey S Wittkowski H Hashkes PJ Berkun Y JAK1/2 inhibition with baricitinib in the treatment of autoinflammatory interferonopathies. J Clin Invest (2018) 128(7):3041–52. 10.1172/JCI98814 29649002 PMC6026004

[51] Zhou Q Wang H Schwartz DM Stoffels M Park YH Zhang Y Loss-of-function mutations in TNFAIP3 leading to A20 haploinsufficiency cause an early-onset autoinflammatory disease. Nat Genet (2016) 48(1):67–73. 10.1038/ng.3459 26642243 PMC4777523

[52] Aeschlimann FA Batu ED Canna SW Go E Gul A Hoffmann P A20 haploinsufficiency (HA20): clinical phenotypes and disease course of patients with a newly recognised NF-kB-mediated autoinflammatory disease. Ann Rheum Dis (2018) 77(5):728–35. 10.1136/annrheumdis-2017-212403 29317407

[53] Berteau F Rouviere B Nau A Le Berre R Sarrabay G Touitou I Response to: ’A20 haploinsufficiency (HA20): clinical phenotypes and disease course of patients with a newly recognised NF-kB-mediated autoinflammatory disease’. Ann Rheum Dis (2018). 10.1136/annrheumdis-2018-213347 29549169

[54] Duncan CJA Dinnigan E Theobald R Grainger A Skelton AJ Hussain R Early-onset autoimmune disease due to a heterozygous loss-of-function mutation in TNFAIP3 (A20). Ann Rheum Dis (2018) 77(5):783–6. 10.1136/annrheumdis-2016-210944 28659290 PMC5909743

[55] Franco-Jarava C Wang H Martin-Nalda A Alvarez SD Garcia-Prat M Bodet D TNFAIP3 haploinsufficiency is the cause of autoinflammatory manifestations in a patient with a deletion of 13Mb on chromosome 6. Clin Immunol (2018) 191:44–51. 10.1016/j.clim.2018.03.009 29572183

[56] Kadowaki T Ohnishi H Kawamoto N Hori T Nishimura K Kobayashi C Haploinsufficiency of A20 causes autoinflammatory and autoimmune disorders. J Allergy Clin Immunol (2018) 141(4):1485–8.e11. 10.1016/j.jaci.2017.10.039 29241730

[57] Ohnishi H Kawamoto N Seishima M Ohara O Fukao T A Japanese family case with juvenile onset Behcet’s disease caused by TNFAIP3 mutation. Allergol Int (2017) 66(1):146–8. 10.1016/j.alit.2016.06.006 27451268

[58] Shigemura T Kaneko N Kobayashi N Kobayashi K Takeuchi Y Nakano N Novel heterozygous C243Y A20/TNFAIP3 gene mutation is responsible for chronic inflammation in autosomal-dominant Behcet’s disease. RMD Open (2016) 2(1):e000223 10.1136/rmdopen-2015-000223 27175295 PMC4860863

[59] Takagi M Ogata S Ueno H Yoshida K Yeh T Hoshino A Haploinsufficiency of TNFAIP3 (A20) by germline mutation is involved in autoimmune lymphoproliferative syndrome. J Allergy Clin Immunol (2017) 139(6):1914–22. 10.1016/j.jaci.2016.09.038 27845235

[60] Viel S Cheyssac E Pescarmona R Besson L Till M Viremouneix L Large deletion in 6q associated to A20 haploinsufficiency and thoracoabdominal heterotaxy. Ann Rheum Dis (2018). 10.1136/annrheumdis-2018-213300 29678940

[61] Ye Z Zhou Y Huang Y Wang Y Lu J Tang Z Phenotype and management of infantile-onset inflammatory bowel disease: experience from a tertiary care center in China. Inflamm Bowel Dis (2017) 23(12):2154–64. 10.1097/MIB.0000000000001269 29140941

[62] Ozen S Batu ED. The myths we believed in familial Mediterranean fever: what have we learned in the past years? Semin Immunopathol (2015) 37(4):363–9. 10.1007/s00281-015-0484-6 25832989

[63] Tunca M Akar S Onen F Ozdogan H Kasapcopur O Yalcinkaya F Familial Mediterranean fever (FMF) in Turkey: results of a nationwide multicenter study. Medicine (2005) 84(1):1–11. 10.1097/01.md.0000152370.84628.0c 15643295

[64] Jain A Misra DP Sharma A Wakhlu A Agarwal V Negi VS. Vasculitis and vasculitis-like manifestations in monogenic autoinflammatory syndromes. Rheumatol Int (2018) 38(1):13–24. 10.1007/s00296-017-3839-6 29032440

[65] Karadag O Jayne DJ. Polyarteritis nodosa revisited: a review of historical approaches, subphenotypes and a research agenda. Clin Exp Rheumatol (2018) 36:135–42. 29465365

[66] Imirzalioglu N Dursun A Tastan B Soysal Y Yakicier MC MEFV gene is a probable susceptibility gene for Behcet’s disease. Scand J Rheumatol (2005) 34(1):56–8. 10.1080/03009740510017931 15903027

[67] Schwartz T Langevitz P Zemer D Gazit E Pras M Livneh A Behcet’s disease in Familial Mediterranean fever: characterization of the association between the two diseases. Semin Arthritis Rheum (2000) 29(5):286–95. 10.1016/S0049-0172(00)80015-3 10805353

[68] Touitou I Magne X Molinari N Navarro A Quellec AL Picco P MEFV mutations in Behcet’s disease. Hum Mutat (2000) 16(3):271–2. 10.1002/1098-1004(200009)16:3<271:AID-HUMU16>3.0.CO;2-A 10980540

[69] Wu Z Zhang S Li J Chen S Li P Sun F Association between MEFV mutations M694V and M680I and Behcet’s disease: a meta-analysis. PLoS One (2015) 10(7):e0132704 10.1371/journal.pone.0132704 26176758 PMC4503748

[70] Biomarkers Definitions Working Group. Biomarkers and surrogate endpoints: preferred definitions and conceptual framework. Clin Pharmacol Ther (2001) 69(3):89–95. 10.1067/mcp.2001.113989 11240971

[71] Sun L Xie B Zhang Q Wang Y Wang X Gao B Biomarkers identification by a combined clinical and metabonomics analysis in Henoch-Schonlein purpura nephritis children. Oncotarget (2017) 8(69):114239–50. 10.18632/oncotarget.23207 29371982 PMC5768399

[72] Berthelot L Jamin A Viglietti D Chemouny JM Ayari H Pierre M Value of biomarkers for predicting immunoglobulin A vasculitis nephritis outcome in an adult prospective cohort. Nephrol Dial Transplant (2017) gfx300. 10.1093/ndt/gfx300 29126311

[73] Ge W Wang HL Sun RP Pentraxin 3 as a novel early biomarker for the prediction of Henoch-Schonlein purpura nephritis in children. Eur J Pediatr (2014) 173(2):213–8. 10.1007/s00431-013-2150-0 23963627

[74] Xu H Li W Mao JH Pan YX. Association between red blood cell distribution width and Henoch-Schonlein purpura nephritis. Medicine (2017) 96(23):e7091. 10.1097/MD.0000000000007091 28591051 PMC5466229

[75] Zhang L Han C Sun C Meng H Ye F Na S Serum levels of alpha-smooth muscle actin and c-Met as biomarkers of the degree of severity of Henoch-Schonlein purpura nephritis. Transl Res (2013) 161(1):26–36. 10.1016/j.trsl.2012.09.001 23041443

[76] Zhou TB Yin SS Association of matrix metalloproteinase-9 level with the risk of renal involvement for Henoch-Schonlein purpura in children. Ren Fail (2013) 35(3):425–9. 10.3109/0886022X.2012.757826 23356642

[77] Jiang J Duan W Shang X Wang H Gao Y Tian P Inducible nitric oxide synthase gene polymorphisms are associated with a risk of nephritis in Henoch-Schonlein purpura children. Eur J Pediatr (2017) 176(8):1035–45. 10.1007/s00431-017-2945-5 28593405

[78] Lopez-Mejias R Genre F Remuzgo-Martinez S Sevilla Perez B Castaneda S Llorca J Interleukin 1 beta (IL1ss) rs16944 genetic variant as a genetic marker of severe renal manifestations and renal sequelae in Henoch-Schonlein purpura. Clin Exp Rheumatol (2016) 34(3 Suppl 97):S84–8. 26842496

[79] Gunasekaran TS Berman J Gonzalez M Duodenojejunitis: is it idiopathic or is it Henoch-Schonlein purpura without the purpura? J Pediatr Gastroenterol Nutr (2000) 30(1):22–8. 10.1097/00005176-200001000-00013 10630435

[80] Kawasaki K Komura H Nakahara Y Shiraishi M Higashida M Ouchi K Factor XIII in Henoch-Schonlein purpura with isolated gastrointestinal symptoms. Pediatr Int (2006) 48(4):413–5. 10.1111/j.1442-200X.2006.02232.x 16911090

[81] Guo MM Tseng WN Ko CH Pan HM Hsieh KS Kuo HC. Th17- and Treg-related cytokine and mRNA expression are associated with acute and resolving Kawasaki disease. Allergy (2015) 70(3):310–8. 10.1111/all.12558 25585854

[82] Yamamoto N Sato K Hoshina T Kojiro M Kusuhara K. Utility of ferritin as a predictor of the patients with Kawasaki disease refractory to intravenous immunoglobulin therapy. Mod Rheumatol (2015) 25(6):898–902. 10.3109/14397595.2015.1038430 25849851

[83] Okuma Y Suda K Nakaoka H Katsube Y Mitani Y Yoshikane Y Serum tenascin-C as a novel predictor for risk of coronary artery lesion and resistance to intravenous immunoglobulin in Kawasaki disease – a multicenter retrospective study. Circ J (2016) 80(11):2376–81. 10.1253/circj.CJ-16-0563 27746411

[84] Sato S Kawashima H Kashiwagi Y Hoshika A. Inflammatory cytokines as predictors of resistance to intravenous immunoglobulin therapy in Kawasaki disease patients. Int J Rheum Dis (2013) 16(2):168–72. 10.1111/1756-185X.12082 23773640

[85] Motoki N Akazawa Y Yamazaki S Hachiya A Motoki H Matsuzaki S Prognostic significance of QT interval dispersion in the response to intravenous immunoglobulin therapy in Kawasaki disease. Circ J (2017) 81(4):537–42. 10.1253/circj.CJ-16-0864 28154289

[86] Ye Q Gong FQ Shang SQ Hu J. Intravenous immunoglobulin treatment responsiveness depends on the degree of CD8+ T cell activation in Kawasaki disease. Clin Immunol (2016) 171:25–31. 10.1016/j.clim.2016.08.012 27519954

[87] Kirchhof MG Lee AY Dutz JP. D-dimer levels as a marker of cutaneous disease activity: case reports of cutaneous polyarteritis nodosa and atypical recurrent urticaria. JAMA Dermatol (2014) 150(8):880–4. 10.1001/jamadermatol.2013.9944 24695854

[88] Navarro M Cervera R Font J Reverter JC Monteagudo J Escolar G Anti-endothelial cell antibodies in systemic autoimmune diseases: prevalence and clinical significance. Lupus (1997) 6(6):521–6. 10.1177/096120339700600608 9256310

[89] Okano T Takeuchi S Soma Y Suzuki K Tsukita S Ishizu A Presence of anti-phosphatidylserine-prothrombin complex antibodies and anti-moesin antibodies in patients with polyarteritis nodosa. J Dermatol (2017) 44(1):18–22. 10.1111/1346-8138.13491 27345569

[90] Fussner LA Hummel AM Schroeder DR Silva F Cartin-Ceba R Snyder MR Factors determining the clinical utility of serial measurements of antineutrophil cytoplasmic antibodies targeting proteinase 3. Arthritis Rheumatol (2016) 68(7):1700–10. 10.1002/art.39637 26882078 PMC7137903

[91] Kemna MJ Damoiseaux J Austen J Winkens B Peters J van Paassen P ANCA as a predictor of relapse: useful in patients with renal involvement but not in patients with nonrenal disease. J Am Soc Nephrol (2015) 26(3):537–42. 10.1681/ASN.2013111233 25324502 PMC4341475

[92] Lionaki S Blyth ER Hogan SL Hu Y Senior BA Jennette CE Classification of antineutrophil cytoplasmic autoantibody vasculitides: the role of antineutrophil cytoplasmic autoantibody specificity for myeloperoxidase or proteinase 3 in disease recognition and prognosis. Arthritis Rheum (2012) 64(10):3452–62. 10.1002/art.34562 23023777 PMC3462364

[93] Murosaki T Sato T Akiyama Y Nagatani K Minota S. Difference in relapse-rate and clinical phenotype by autoantibody-subtype in Japanese patients with anti-neutrophil cytoplasmic antibody-associated vasculitis. Mod Rheumatol (2017) 27(1):95–101. 10.1080/14397595.2016.1192760 27320904

[94] Schirmer JH Wright MN Herrmann K Laudien M Nolle B Reinhold-Keller E Myeloperoxidase-antineutrophil cytoplasmic antibody (ANCA)-positive granulomatosis with polyangiitis (Wegener’s) is a clinically distinct subset of ANCA-associated vasculitis: a retrospective analysis of 315 patients from a German vasculitis referral center. Arthritis Rheumatol (2016) 68(12):2953–63. 10.1002/art.39786 27333332

[95] Kemna MJ Plomp R van Paassen P Koeleman CAM Jansen BC Damoiseaux J Galactosylation and sialylation levels of IgG predict relapse in patients with PR3-ANCA associated vasculitis. EBioMedicine (2017) 17:108–18. 10.1016/j.ebiom.2017.01.033 28169190 PMC5360573

[96] Ishizaki J Takemori A Suemori K Matsumoto T Akita Y Sada KE Targeted proteomics reveals promising biomarkers of disease activity and organ involvement in antineutrophil cytoplasmic antibody-associated vasculitis. Arthritis Res Ther (2017) 19(1):218. 10.1186/s13075-017-1429-3 28962592 PMC5622475

[97] Rani L Minz RW Arora A Kannan M Sharma A Anand S Serum proteomic profiling in granumomatosis with polyangiitis using two-dimensional gel electrophoresis along with matrix assisted laser desorption ionization time of flight mass spectrometry. Int J Rheum Dis (2014) 17(8):910–9. 10.1111/1756-185X.12481 25350587

[98] Stone JH Rajapakse VN Hoffman GS Specks U Merkel PA Spiera RF A serum proteomic approach to gauging the state of remission in Wegener’s granulomatosis. Arthritis Rheum (2005) 52(3):902–10. 10.1002/art.20938 15751091

[99] McKinney EF Lyons PA Carr EJ Hollis JL Jayne DR Willcocks LC A CD8+ T cell transcription signature predicts prognosis in autoimmune disease. Nat Med (2010) 16(5):586–91, 1p following 91. 10.1038/nm.2130 20400961 PMC3504359

[100] McKinney EF Lee JC Jayne DR Lyons PA Smith KG T-cell exhaustion, co-stimulation and clinical outcome in autoimmunity and infection. Nature (2015) 523(7562):612–6. 10.1038/nature14468 26123020 PMC4623162

[101] Unizony S Villarreal M Miloslavsky EM Lu N Merkel PA Spiera R Clinical outcomes of treatment of anti-neutrophil cytoplasmic antibody (ANCA)-associated vasculitis based on ANCA type. Ann Rheum Dis (2016) 75(6):1166–9. 10.1136/annrheumdis-2015-208073 26621483 PMC4908815

[102] Haubitz M Good DM Woywodt A Haller H Rupprecht H Theodorescu D Identification and validation of urinary biomarkers for differential diagnosis and evaluation of therapeutic intervention in anti-neutrophil cytoplasmic antibody-associated vasculitis. Mol Cell Proteomics (2009) 8(10):2296–307. 10.1074/mcp.M800529-MCP200 19564150 PMC2758757

[103] Hong Y Eleftheriou D Hussain AA Price-Kuehne FE Savage CO Jayne D Anti-neutrophil cytoplasmic antibodies stimulate release of neutrophil microparticles. J Am Soc Nephrol (2012) 23(1):49–62. 10.1681/ASN.2011030298 22052057 PMC3269928

[104] Gomez-Moreno D Adrover JM Hidalgo A. Neutrophils as effectors of vascular inflammation. Eur J Clin Invest (2018):e12940. 10.1111/eci.12940 29682731

[105] Kessenbrock K Krumbholz M Schonermarck U Back W Gross WL Werb Z Netting neutrophils in autoimmune small-vessel vasculitis. Nat Med (2009) 15(6):623–5. 10.1038/nm.1959 19448636 PMC2760083

[106] Wang H Sha LL Ma TT Zhang LX Chen M Zhao MH Circulating level of neutrophil extracellular traps is not a useful biomarker for assessing disease activity in antineutrophil cytoplasmic antibody-associated vasculitis. PLoS One (2016) 11(2):e0148197 10.1371/journal.pone.0148197 26840412 PMC4739550

[107] Deshayes S Aouba A Khoy K Mariotte D Lobbedez T Martin Silva N. Hypocomplementemia is associated with worse renal survival in ANCA-positive granulomatosis with polyangiitis and microscopic polyangiitis. PLoS One (2018) 13(4):e0195680. 10.1371/journal.pone.0195680 29621352 PMC5886583

[108] Jayne DRW Bruchfeld AN Harper L Schaier M Venning MC Hamilton P Randomized trial of C5a receptor inhibitor avacopan in ANCA-associated vasculitis. J Am Soc Nephrol (2017) 28(9):2756–67. 10.1681/ASN.2016111179 28400446 PMC5576933

[109] Becker H Maaser C Mickholz E Dyong A Domschke W Gaubitz M. Relationship between serum levels of macrophage migration inhibitory factor and the activity of antineutrophil cytoplasmic antibody-associated vasculitides. Clin Rheumatol (2006) 25(3):368–72. 10.1007/s10067-005-0045-9 16391884

[110] Kim HJ Jung SM Song JJ Park YB Lee SW. Mean platelet volume can estimate the current vasculitis activity of microscopic polyangiitis. Rheumatol Int (2018) 38(6):1095–101. 10.1007/s00296-018-4011-7 29556749

[111] Kucuk H Varan O Goker B Bitik B Ozturk MA Haznedaroglu S Serum ferritin as an activity marker for granulamotosis with polyangiitis. Ren Fail (2017) 39(1):566–9. 10.1080/0886022X.2017.1349675 28741986 PMC6446145

[112] Watanabe S Gono T Nishina K Sugitani N Watanabe E Yabe H Rheumatoid factor is correlated with disease activity and inflammatory markers in antineutrophil cytoplasmic antibody-associated vasculitis. BMC Immunol (2017) 18(1):53. 10.1186/s12865-017-0234-8 29262790 PMC5738867

[113] Yoo J Ahn SS Jung SM Song JJ Park YB Lee SW. Delta neutrophil index is associated with vasculitis activity and risk of relapse in ANCA-associated vasculitis. Yonsei Med J (2018) 59(3):397–405. 10.3349/ymj.2018.59.3.397 29611402 PMC5889992

[114] Dagna L Salvo F Tiraboschi M Bozzolo EP Franchini S Doglioni C Pentraxin-3 as a marker of disease activity in Takayasu arteritis. Ann Intern Med (2011) 155(7):425–33. 10.7326/0003-4819-155-7-201110040-00005 21969341

[115] Matsuyama A Sakai N Ishigami M Hiraoka H Kashine S Hirata A Matrix metalloproteinases as novel disease markers in Takayasu arteritis. Circulation (2003) 108(12):1469–73. 10.1161/01.CIR.0000090689.69973.B1 12952836

[116] Noguchi S Numano F Gravanis MB Wilcox JN. Increased levels of soluble forms of adhesion molecules in Takayasu arteritis. Int J Cardiol (1998) 66(Suppl 1):S23–33. 10.1016/S0167-5273(98)00145-4 9951800

[117] Noris M Daina E Gamba S Bonazzola S Remuzzi G. Interleukin-6 and RANTES in Takayasu arteritis: a guide for therapeutic decisions? Circulation (1999) 100(1):55–60. 10.1161/01.CIR.100.1.55 10393681

[118] Arnaud L Haroche J Mathian A Gorochov G Amoura Z. Pathogenesis of Takayasu’s arteritis: a 2011 update. Autoimmun Rev (2011) 11(1):61–7. 10.1016/j.autrev.2011.08.001 21855656

[119] Mahajan N Dhawan V Mahmood S Malik S Jain S. Extracellular matrix remodeling in Takayasu’s arteritis: role of matrix metalloproteinases and adventitial inflammation. Arch Med Res (2012) 43(5):406–10. 10.1016/j.arcmed.2012.07.007 22868188

[120] Sun Y Ma L Yan F Liu H Ding Y Hou J MMP-9 and IL-6 are potential biomarkers for disease activity in Takayasu’s arteritis. Int J Cardiol (2012) 156(2):236–8. 10.1016/j.ijcard.2012.01.035 22330005

[121] Tamura N Maejima Y Tezuka D Takamura C Yoshikawa S Ashikaga T Profiles of serum cytokine levels in Takayasu arteritis patients: potential utility as biomarkers for monitoring disease activity. J Cardiol (2017) 70(3):278–85. 10.1016/j.jjcc.2016.10.016 27989502

[122] Tripathy NK Sinha N Nityanand S. Interleukin-8 in Takayasu’s arteritis: plasma levels and relationship with disease activity. Clin Exp Rheumatol (2004) 22(6 Suppl 36):S27–30. 15675131

[123] Batu ED Sonmez HE Hazirolan T Ozaltin F Bilginer Y Ozen S. Tocilizumab treatment in childhood Takayasu arteritis: case series of four patients and systematic review of the literature. Semin Arthritis Rheum (2017) 46(4):529–35. 10.1016/j.semarthrit.2016.07.012 27515155

[124] Comarmond C Plaisier E Dahan K Mirault T Emmerich J Amoura Z Anti TNF-alpha in refractory Takayasu’s arteritis: cases series and review of the literature. Autoimmun Rev (2012) 11(9):678–84. 10.1016/j.autrev.2011.11.025 22155781

[125] Goel R Nair A Kabeerdoss J Mohan H Jeyaseelan V Joseph G Study of serial serum myeloid-related protein 8/14 as a sensitive biomarker in Takayasu arteritis: a single centre study. Rheumatol Int (2018) 38(4):623–30. 10.1007/s00296-017-3881-4 29196802

[126] Goel R Kabeerdoss J Mohan H Danda S Jayaseelan V Kumar TS Soluble-HLA-E: a follow up biomarker in Takayasu arteritis, independent of HLA-E genotype. Int J Rheum Dis (2018) 21(2):532–40. 10.1111/1756-185X.13027 28425192

[127] Kasuya K Hashimoto Y Numano F. Left ventricular dysfunction and HLA Bw52 antigen in Takayasu arteritis. Heart Vessels Suppl (1992) 7:116–9. 10.1007/BF01744556 1360956

[128] Sadeghi A Davatchi F Shahram F Karimimoghadam A Alikhani M Pezeshgi A Serum profiles of cytokines in Behcet’s disease. J Clin Med (2017) 6(5):49 10.3390/jcm6050049 PMC5447940 28468317

[129] Tulunay A Dozmorov MG Ture-Ozdemir F Yilmaz V Eksioglu-Demiralp E Alibaz-Oner F Activation of the JAK/STAT pathway in Behcet’s disease. Genes Immun (2015) 16(2):170–5. 10.1038/gene.2014.64 25410656 PMC4443896

[130] Morton LT Situnayake D Wallace GR Genetics of Behcet’s disease. Curr Opin Rheumatol (2016) 28(1):39–44. 10.1097/BOR.0000000000000234 26599381

[131] Akman-Demir G Tuzun E Icoz S Yesilot N Yentur SP Kurtuncu M Interleukin-6 in neuro-Behcet’s disease: association with disease subsets and long-term outcome. Cytokine (2008) 44(3):373–6. 10.1016/j.cyto.2008.10.007 19010690

[132] Borhani Haghighi A Ittehadi H Nikseresht AR Rahmati J Poorjahromi SG Pourabbas B CSF levels of cytokines in neuro-Behcet’s disease. Clin Neurol Neurosurg (2009) 111(6):507–10. 10.1016/j.clineuro.2009.02.001 19303205

[133] Gholijani N Ataollahi MR Samiei A Aflaki E Shenavandeh S Kamali-Sarvestani E. An elevated pro-inflammatory cytokines profile in Behcet’s disease: a multiplex analysis. Immunol Lett (2017) 186:46–51. 10.1016/j.imlet.2016.12.001 27939191

[134] Hamzaoui K Hamza M Ayed K Production of TNF-alpha and IL-1 in active Behcet’s disease. J Rheumatol (1990) 17(10):1428–9. 2254912

[135] Lopalco G Lucherini OM Lopalco A Venerito V Fabiani C Frediani B Cytokine signatures in mucocutaneous and ocular Behcet’s disease. Front Immunol (2017) 8:200 10.3389/fimmu.2017.00200 28289419 PMC5327443

[136] Na SY Park MJ Park S Lee ES Up-regulation of Th17 and related cytokines in Behcet’s disease corresponding to disease activity. Clin Exp Rheumatol (2013) 31(3 Suppl 77):32–40. 24064012

[137] Pay S Erdem H Pekel A Simsek I Musabak U Sengul A Synovial proinflammatory cytokines and their correlation with matrix metalloproteinase-3 expression in Behcet’s disease. Does interleukin-1beta play a major role in Behcet’s synovitis? Rheumatol Int (2006) 26(7):608–13. 10.1007/s00296-005-0040-0 16205926

[138] Alibaz-Oner F Sawalha AH Direskeneli H Management of Behcet’s disease. Curr Opin Rheumatol (2018) 30(3):238–42. 10.1097/BOR.0000000000000497 29432223

